# OSMRβ mutants enhance basal keratinocyte differentiation via inactivation of the STAT5/KLF7 axis in PLCA patients

**DOI:** 10.1007/s13238-020-00818-3

**Published:** 2021-01-27

**Authors:** Jun Liu, Junchen Chen, Yadan Zhong, Xiaoling Yu, Ping Lu, Jianqi Feng, Xin Zhang, Shufeng Ma, Chao Yang, Bin Yang, Zhili Rong

**Affiliations:** 1 Dermatology Hospital, Southern Medical University, Guangzhou, 510091 China; 2 Cancer Research Institute, School of Basic Medical Sciences, Southern Medical University, Guangzhou, 510515 China; 3 Bioland Laboratory (Guangzhou Regenerative Medicine and Health Guangdong Laboratory), Guangzhou, 510005 China

**Dear Editor,**

Primary localized cutaneous amyloidosis (PLCA) is a skin-limited disorder characterized by deposition of amyloid material in the superficial dermis. According to clinical characteristics, PLCA is divided into lichen, macular, and nodular amyloidosis. PLCA is found worldwide but has a higher incidence in South America and Southeast Asia, such as in Brazil and China (Chang et al., 2014; Tey et al., 2016). The etiology of PLCA is complicated, involving environmental factors, the immune state, and genetic factors (Tanaka et al., 2009; Katayama et al., 2019). A genome-wide scan revealed that mutations in several genes are involved in the development of PLCA, including oncostatin M receptor (*OSMR*) (Arita et al., 2008), interleukin 31 receptor A (*IL-31RA*) (Shiao et al., 2013), and glycoprotein Nmb (*GPNMB*) (Yang et al., 2018).

Recently, we demonstrated that the c.1538G>A (p.G513D) and c.2081C>T (p.P694L) mutations of *OSMR* were the most frequent mutations in a Chinese PLCA population (Lu et al., 2019). It has been reported that OSM maintains hair follicle stem cell and muscle stem cell quiescence by binding to heterodimeric receptors comprising gp130 and OSMRβ (Sampath et al., 2018; Wang et al., 2019). Additionally, OSM signaling plays crucial roles in the regulation of cardiomyocyte differentiation and cellular plasticity (Kubin et al., 2011). Whether OSMRβ-mediated cell differentiation plays a role in PLCA remains unexplored.

To answer this question, we compared the RNA expression profiles between PLCA patients and healthy controls. Interestingly, Gene Ontology (GO) analysis of the dysregulated genes revealed that most of them were associated with keratinocyte differentiation processes (Fig. 1A). Consistent with the above findings, in PLCA patients with *OSMR* mutations, epidermal keratinocyte differentiation was enhanced, with increased expression of FLG and LOR, compared to that in healthy controls (Fig. 1B). Furthermore, immunofluorescence analysis suggests that the expression of Ki67, an indicator of cell proliferation, was also enhanced in the epidermis of PLCA patients with *OSMR* mutations (Fig. 1C and 1D).

**Figure 1:**
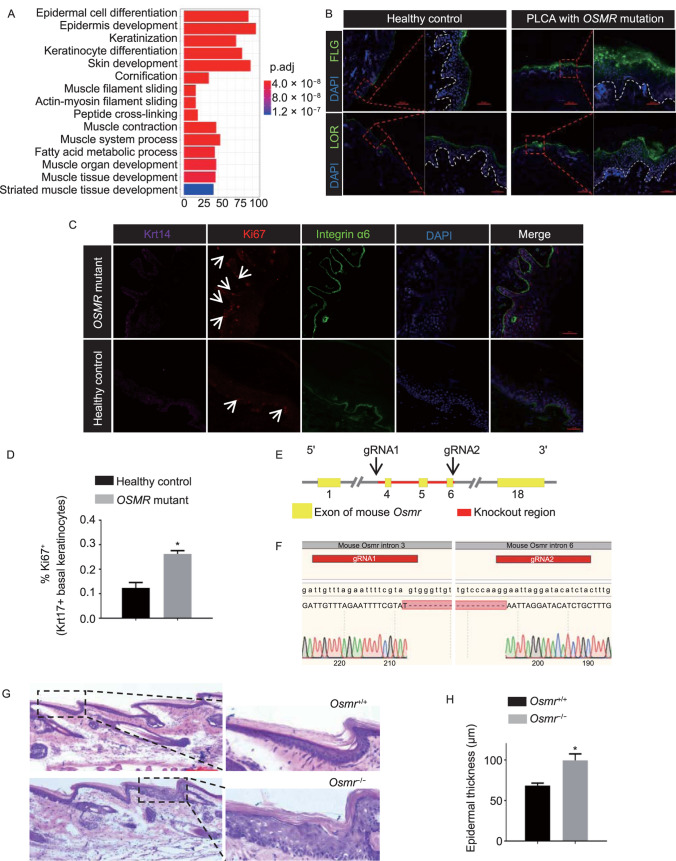

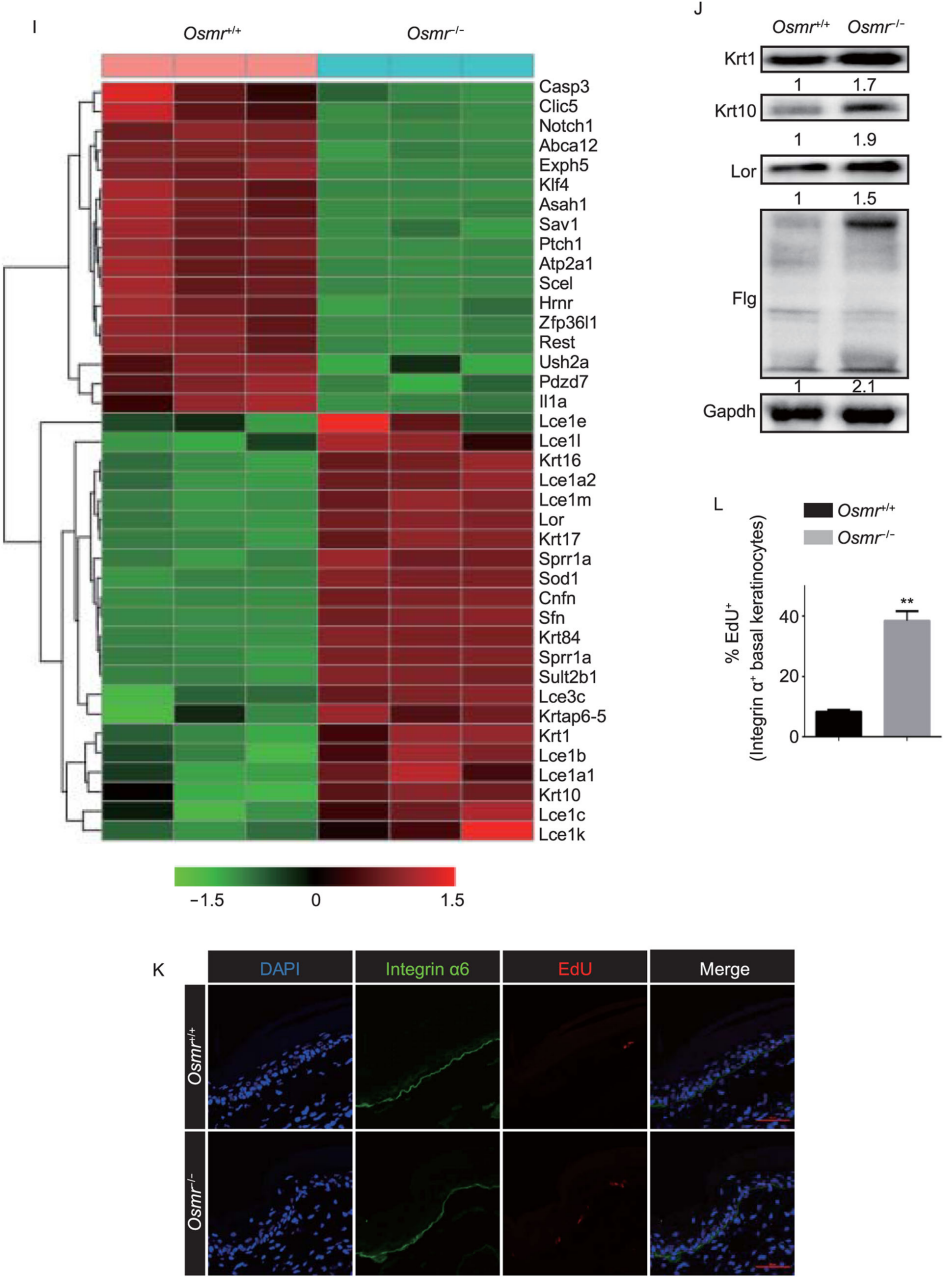
Enhanced differentiation and proliferation of basal keratinocytes in PLCA patients with *OSMR* mutations and in *Osmr* ^−/−^ **mice.** (A) Top-ranked enriched Gene Ontology (GO) biological processes in PLCA patients with *OSMR* mutations versus healthy controls. (B) Immunofluorescence analysis showing increased expression of FLG and LOR in PLCA skin lesion. Scale bars, 200 µm and 50 µm (inset). (C) Immunofluorescence analysis showing increased expression of Ki67 in basal keratinocytes of PLCA skin lesion. Scale bars, 50 µm. (D) Quantification of Ki67^+^ basal keratinocytes. Data are mean ± SD from three independent experiments. *P < 0.05, one-way analysis of variance (ANOVA). (E) Schematic of knockout strategy for *Osmr*^−/−^ mice. (F) Verification of *Osmr*^−/−^ mice using Sanger sequencing. (G) Hematoxylin & Eosin staining of WT and *Osmr*^−/−^ mice tail skin. Representative images of three litters (post-natal day 30 (P30); *n* = 19, males = 10, females = 9) with 5–8 mice per litter. Scale bar, 100 μm. (H) Quantification of epidermal thickness on P30. (I) Hierarchical clustering heatmap of differential expression of keratinocyte differentiation-related genes in dorsal epidermis from WT and *Osmr*^−/−^ mice. (J) Western blot analysis of keratinocyte differentiation-related proteins using lysate of WT and *Osmr*^−/−^ mice skin. Representative images of three biological replicates. (K) 5-ethynyl-2’-deoxyuridine (EdU) incorporation analysis. Integrin α6 and EdU were stained in tail skin from WT and *Osmr*^−/−^ mice 48 h after EdU administration. Scale bar, 50 μm. (L) Quantification of EdU^+^ basal keratinocytes. Data are mean ± SD from three independent experiments. **P < 0.01, one-way analysis of variance (ANOVA)

To further determine the biological functions of OSMRβ protein in the skin, *Osmr*^−/−^ C57BL/6 mice were produced using the CRISPR/Cas9 system (Figs. 1E, 1F and S1A). Unfortunately, no PLCA-like phenotype was observed in these mice under physiological or pathological conditions (including UVA exposure and an itch challenge; data not shown). Hair follicle cycle changes were observed between WT and *Osmr*^−/−^ mice at post-natal day 30 (P30) using hematoxylin and eosin staining (Fig. S1B), which is consistent with previous reports (Wang et al., 2019). More importantly, the tail epidermal thickness was significantly increased in *Osmr*^−/−^ mice compared to WT mice (Fig. 1G and 1H). RNA-seq analysis indicated a 2-fold change in the expression levels of 2,328 genes in the skin of *Osmr*^−/−^ mice compared to their WT littermates. GO analysis showed that the genes related to certain functions, such as keratinocyte differentiation and skin development, were dysregulated, which suggests that epidermal keratinocyte differentiation was enhanced in the skin of *Osmr*^−/−^ mice (Fig. S1C). Of the differentially expressed genes between the two groups, 39 genes are known to be related to epidermal keratinocyte differentiation (Fig. 1I). To validate our findings, we used qRT-PCR to examine terminal differentiation marker expression in freshly isolated skin from *Osmr*^−/−^ mice and their WT littermates. This analysis confirmed increasing expression levels of *Krt1, Krt10, Flg* and *Lor* in skin from *Osmr*^−/−^ mice (Fig. S1D). The different expression level of proteins was confirmed by Western blot (Fig. 1J). We next explored whether *Osmr* knockout affects basal keratinocyte proliferation. The EdU incorporation results showed that basal keratinocyte proliferation was significantly increased in both the tail (Fig. 1K and 1L) and dorsal (Fig. S1E and S1F) skin of the *Osmr*^−/−^ mice compared to their WT littermates. These results suggest that *Osmr* knockout enhances basal keratinocyte differentiation and proliferation in mice.

OSMRβ is a component of both the OSM type II receptor and the IL-31 receptor, so we sought to investigate which of these cytokines was involved in the regulation of human keratinocyte differentiation. HaCaT and primary keratinocytes were cultured and stimulated with indicated concentration of OSM or IL-31, and the expression levels of epidermal keratinocyte differentiation-related genes were significantly decreased in the OSM-treated HaCaT cells or primary keratinocytes (Figs. 2A, 2B and S2A–D). Further, we validated our results using 3D skin models. Consistently, qRT-PCR and immunofluorescence analysis demonstrated the decreased expression of epidermal keratinocyte differentiation markers in OSM-treated 3D skins (Figs. 2C and S2E).

**Figure 2:**
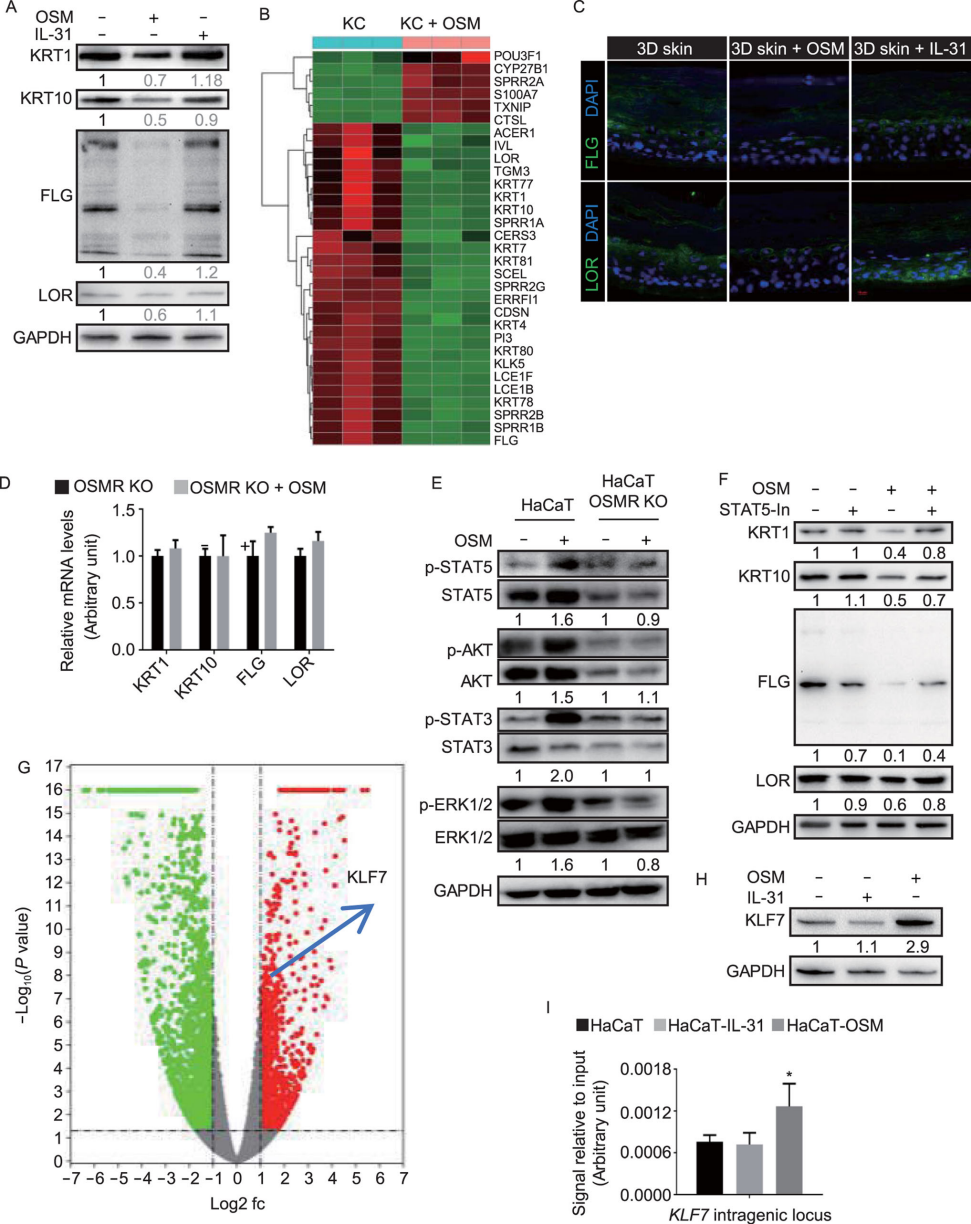

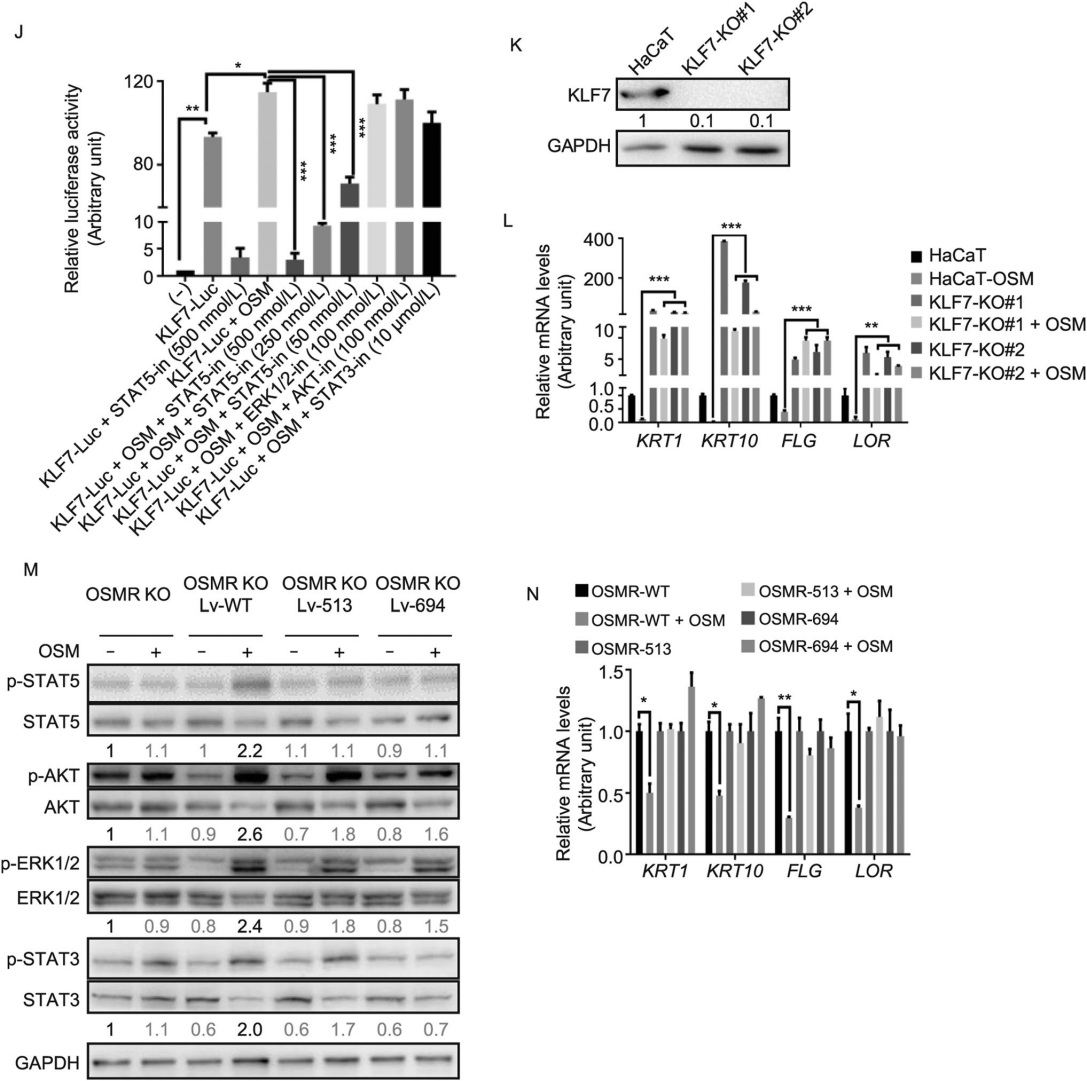
OSMRβ mutants enhance keratinocyte differentiation via losing the function to activate the OSM/OSMRβ/STAT5/KLF7 axis. (A) OSM inhibits KRT1, KRT10, FLG, and LOR expression. Western blot of HaCaT cells stimulated with 10 ng/mL OSM or 100 ng/mL IL-31 for 72 h. (B) Hierarchical clustering heatmap of differential expression of keratinocyte differentiation-related genes in primary keratinocytes with or without OSM stimulation. (C) Immunofluorescence analysis showing decreased expression of FLG and LOR in OSM-treated 3D skin. Scale bars, 10 µm. (D) Knockout of *OSMR* in HaCaT cells ameliorates the OSM-induced expression decrease of keratinocyte differentiation-related genes. (E) Western blot of indicated proteins in HaCaT cells and OSMR-knockout HaCaT cells stimulated with 10 ng/mL OSM for 15 min. (F) Pretreatment with a STAT5 inhibitor rescues the OSM-induced decreased expression of keratinocyte differentiation-related genes. (G) Volcano plot comparing the *p*_*adj*_ value versus fold change for HaCaT cells treated with or without OSM. Green and red dots represent genes with a *p*_*adj*_ < 0.05 and >2-fold change. (H) Western blot of OSM-treated HaCaT cells showing increased expression of KLF7 protein. (I) ChIP-qPCR analysis with an anti-STAT5 antibody in HaCaT cells treated with IL-31 or OSM. (J) Luciferase reporter assay of the *KLF7* promoter showing the requirement of STAT5 activation for OSM-induced KLF7 expression. (K) Western blot showing no KLF7 expression in two independent *KLF7*-knockout HaCaT cell clones. (L) *KLF7* knockout rescues the OSM-induced decreased expression of keratinocyte differentiation-related genes. (M) *OSMR* p.G513D and p.P694L are partial loss-of-function mutants. Western blot of *OSMR*-knockout HaCaT cells transduced with indicated OSMR mutants. (N) Both *OSMR* p.G513D and p.P694L mutations rescue the OSM-induced decreased expression of keratinocyte differentiation-related genes. For (A, E, F, H, K, and M), representative images of three biological replicates are shown, and quantification of each immunoblotting was labeled under each band (Online methods for detailed information); For (D, I, J, L, and N), data are mean ± SEM, *n* = 3, *P < 0.05, **P < 0.01, ***P < 0.001, one-way analysis of variance (ANOVA)

To further confirm that OSM inhibits keratinocyte differentiation through heterodimeric receptors comprising gp130 and OSMRβ, the CRISPR/Cas9 system was employed to produce *OSMR-*knockout HaCaT cells (Fig. S2F–H). The results indicated that knockout of *OSMR* can rescue OSM-induced inhibition of keratinocyte differentiation (Fig. 2D). Next, we aimed to identify the molecular mechanisms underlying OSM-induced inhibition of keratinocyte differentiation. Western blot showed that HaCaT cells exhibited activation of STAT3, STAT5, ERK1/2, and AKT signaling after OSM stimulation (Fig. S2I). However, only the STAT3 and ERK1/2 pathways were activated in IL-31-treated HaCaT cells (Fig. S2I). We further demonstrated that *OSMR* knockout can block OSM-induced phosphorylation of STAT3, STAT5, ERK1/2, and AKT in HaCaT cells (Fig. 2E). In an attempt to delineate the downstream signaling pathways, HaCaT cells were pretreated with inhibitors before being stimulated with OSM. STAT5 inhibitor could almost completely rescue the decreased mRNA expression of keratinocyte differentiation-related genes (Fig. S2J). STAT3 inhibitor had a partial rescue effect (Fig. S2K). And ERK1/2 (Fig. S2L) and AKT (Fig. S2M) inhibitors had no effect at all. Similar results were observed when the protein express levels of these differentiation genes were checked (Figs. 2F and S2N). These findings strongly suggest that OSM/OSMRβ signaling, likely via JAK/STAT5, is involved in the regulation of keratinocyte differentiation.

Next, we tried to identify critical factors downstream STAT5 to regulate keratinocyte differentiation. An increased abundance of *KLF7* (Fig. 2G), a transcription factor involved in the regulation of somatic stem cell quiescence (Wang et al., 2016), was observed in OSM-treated HaCaT cells. In contrast, decreased *Kif7* expression was found in *Osmr*^−/−^ mice compared to WT mice (Fig. S3A). OSM-induced upregulation of this gene in mRNA and protein in HaCaT cells was further identified using qRT-PCR and Western blot, respectively (Figs. 2H and S3B).

Further, we analyzed two available STAT5 ChIP-seq data sets involving human B lymphocytes (Gertz et al., 2013) and mice natural killer cells (Villarino et al., 2017). The results indicated that human and mice STAT5-binding sites at the *KLF7* intragenic locus are highly conserved (Fig. S3C and S3D). Furthermore, ChIP-qPCR confirmed that STAT5 binds to the *KLF7* gene locus in human keratinocytes upon OSM stimulation (Fig. 2I). In addition, we cloned approximately 2 kb of the upstream region of the *KLF7* gene transcriptional start site (defined as *KLF7* promoter in our study) into a pGL4 luciferase reporter vector, and we then tested whether the luciferase reporter activity is regulated by STAT5 in HEK293T cells. As shown in Fig. 2J, the upregulation of luciferase activity in OSM-stimulated HEK293T cells was inhibited, in a dose-dependent manner, by pretreatment with a STAT5 inhibitor, but not by pretreatment with STAT3, ERK1/2, or AKT inhibitors. Three potential STAT5-binding sites (ChIP-seq peaks) were found within the *KLF7* promoter region, combinational deletion experiments demonstrated that all the three sites contributed to *KLF7* expression (Fig. S3E). These above data indicate that *KLF7* is a direct target gene of STAT5.

To investigate the function of KLF7 in keratinocyte differentiation, KLF7-overexpressing lentivirus was packaged and transduced into HaCaT cells. As expected, qRT-PCR and Western blot revealed that the expression levels of KRT1, KRT10, FLG, and LOR were decreased in KLF7-overexpressing HaCaT cells (Fig. S3F and S3G). Two independent siRNAs resulted in efficient knockdown of KLF7 (Fig. S3H) and in a reduction of OSM-induced keratinocyte differentiation (Fig. S3I). To further confirm our results, CRISPR/Cas9 technology was employed to generate *KLF7*-knockout HaCaT cell lines (Fig. 2K). Loss of KLF7 also resulted in the inhibition of OSM-induced keratinocyte differentiation (Fig. 2L). These findings support our hypothesis that OSM inhibits keratinocyte differentiation through activation of the STAT5/KLF7 signaling pathway.

Several studies have demonstrated that missense mutations of *OSMR* are involved in the development of PLCA (Arita et al., 2008; Wali et al., 2015). However, the changes in the biological function of the OSMRβ protein caused by the mutations still need to be identified. The *OSMR*-knockout HaCaT cell line was infected with Lv-OSMR-pG513D-P2A-GFP, Lv-OSMR-pP694L-P2A-GFP, or the Lv-OSMR-WT-P2A-GFP virus as control (Fig. S3J). We assessed whether the p.G513D and p.P694L variants resulted in mislocalization of OSMRβ using immunofluorescence analysis. The results showed that the cellular localization of WT-OSMRβ and the two variants (with the p.G513D and p.P694L mutations) were similar, indicating that neither variant impacted the overall cellular localization (Fig. S3K). We next checked the downstream signaling pathways activated by the two variants. Western blot showed that p.P694L mutant failed to activate STAT5 and STAT3, and that p.G513D mutant failed to activate STAT5 (Fig. 2M). No dominant negative effect was observed, as OSM can activate either STAT5 or STAT3 phosphorylation in both WT/p.G513D and WT/p.P694L co-infected HaCaT cells (Fig. S3L). More importantly, qRT-PCR analysis further confirmed that the *OSMR* mutations resulted in the inhibition of OSM-induced keratinocyte differentiation (Fig. 2N).

In summary, we identified OSM as a negative regulator of epidermal keratinocyte differentiation that acts via STAT5/KLF7 signaling *in vivo* and *in vitro*. Dysregulation of the OSM/OSMRβ/STAT5/KLF7 axis by *OSMR* mutation could lead to PLCA (Fig. S4). Therefore, this study discovered the potential underlying cellular and molecular mechanisms how *OSMR* mutations caused PLCA, and the discovered STAT5/KLF7 molecule axis could be a potential target for PLCA treatment in the future.

## FOOTNOTES

We thank all members of the Rong and Yang labs for their helpful discussions and suggestions regarding the manuscript. The research was funded by the National Natural Science Foundation of China (82003329, 82073418, 81872511 and 81670093), Frontier Research Program of Bioland Laboratory (Guangzhou Regenerative Medicine and Health Guangdong Laboratory) (2018GZR110105005), National Science and Technology Major Project (2018ZX10301101), Guangdong Basic and Applied Basic Research Foundation (2019A1515110109), and China Postdoctoral Science Foundation (2019M662983).

The accession numbers for the RNA sequencing data reported in this paper are GEO: GSE150884, GSE150994 and GSE151174.

Jun Liu, Junchen Chen, Yadan Zhong, Xiaoling Yu, Jianqi Feng, Xin Zhang, Shufeng Ma, Chao Yang, Bin Yang, and Zhili Rong declare that they have no conflict of interest. All procedures followed were in accordance with the ethical standards of the responsible committee on human experimentation (the Dermatology Hospital of Southern Medical University) and with the Helsinki Declaration of 1975, as revised in 2000 (5). Informed consent was obtained from all patients for being included in the study. All institutional and national guidelines for the care and use of laboratory animals were followed.

## Supplementary information

Below is the link to the electronic supplementary material.

Supplementary material 1 (PDF 694 kb)
