# Supplementary material for: OSMRβ mutants enhance basal keratinocyte differentiation via inactivation of the STAT5/KLF7 axis in PLCA patients
Source: Protein Cell. 2021 Jan 27;12(8):653–61. doi: 10.1007/s13238-020-00818-3 (PMC8310549; doi:10.1007/s13238-020-00818-3)
Supplement: Supplementary file 1 — Supplementary material 1 (PDF 694 kb) [file 13238_2020_818_MOESM1_ESM.pdf]

METHODS

Subjects and experimental animals

After obtaining written informed consent from the participants and the approval of the Ethics Committee of the Dermatology Hospital of Southern Medical University, we collected 4–5ml peripheral venous blood and the corresponding clinical information from the PLCA patients attending the hospital and their family members who also had PLCA. All the processes were in line with the Helsinki Declaration. We then extracted the genomic DNA from the peripheral blood using blood DNA kits (DP314; TIANGEN Biotech, China). We designed primers for the coding sequence of the *OSMR* gene (GenBank accession number: NM_003999.3) using Primer blast at NCBI, and the primers were synthesized by BGI Group (Beijing, China) (Supplemental Table 1). After PCR, the products were purified and sequenced.

All mice used in this study were with the C57BL/6 background. *Osmr*-knockout ($Osmr^{-/-}$) C57BL/6 mice were obtained from Cyagen Biosciences (KOCMP-00202-Osmr; Guangzhou, China). The mice were bred and maintained in a specific-pathogen-free facility in accordance with the guidelines of the Laboratory Animal Resource Center and the Institutional Animal Care and Use Committee of the Southern Medical University. The mice were housed under a 12 h light/dark cycle.

Cell lines and culture conditions

Immortalized human HaCaT keratinocytes (RRID:CVCL_0038) and human embryonic kidney HEK293T cells (ATCC: CRL-11268) were maintained in Dulbecco's Modified Eagle Medium (DMEM; C11995500CP; Gibco, USA) supplemented with 10% (V/V) fetal bovine serum (FBS; A31608-02; Gibco, USA), 100 units/ml penicillin, and 100 mg/ml streptomycin (15140-122; Invitrogen, USA). Primary keratinocytes were isolated from the foreskins of newborn babies, as previously described (Johansen, 2017), and maintained in

defined keratinocyte serum-free medium (10744019; Thermo Fisher, USA). The cells were cultured at 37°C in a humidified atmosphere with 5% CO_2 in air.

3D skin culture

3D skins (EpiKutis®, PM1011) were purchased from Guangdong Biocell Biothechnology Co. LTD. 3D model were culture in EpiGrowth medium (PY3021) according to the manufacturer's protocol.

Inhibitors, cytokines, and plasmids

Inhibitors of STAT5 (cenisertib; HY-13072), AKT (AZD5363; HY-15431), and ERK1/2 (ravoxertinib hydrochloride; HY-15947A) were purchased from MedChemExpress (USA). Inhibitor of STAT3 (static; S7024) was purchased from Selleck (China). All inhibitors were dissolved in dimethyl sulfoxide (DMSO; D2650; Sigma, USA). Human OSM (295-OM-010) and human IL-31 (2824-IL-010) were purchased from R&D Systems (USA). All cytokines were dissolved in sterile phosphate-buffered saline (PBS).

The cDNA fragments of human wildtype (WT) *OSMR* (*h*OSMR-WT) and human *OSMR* with the p.G513D or p.P694L mutation (*h*OSMR-pG513D and *h*OSMR-pP694L, respectively) were chemically synthesized (BGI, China). A myc tag coding region was fused to the 5' end, after the signal peptide, of the WT and mutated *OSMR* cDNA fragments. The cDNA fragment of human *KLF7* was obtained by reverse transcription (RT)-PCR using HaCaT cell mRNA as the template. A 3×flag coding region was fused to the 5' end of the *KLF7* cDNA fragment.

Lentiviral overexpression plasmids were constructed as described previously (Liu et al., 2018). The resulting plasmids were designated pLv-OSMR-WT-P2A-GFP, pLv-OSMR-pG513D-P2A-GFP, pLv-OSMR-pP694L-P2A-GFP, and pLv-KLF7-IRES-GFP. Single guide RNAs (sgRNAs) targeting the human and mice *OSMR* and human *KLF7* genes were designed using the Zhang Lab clustered regularly interspaced short

palindromic repeats (CRISPR) design algorithm (https://sg.idtdna.com/site/order/designtool/ index/CRISPR_CUSTOM). The DNA target sequences of these sgRNAs are listed in Supplemental Table 1. DNA oligos encoding sgRNAs were cloned into pU6-sgRNA-scaffold2.1, as previously described (Huang et al., 2020). Approximately 2 kb of the upstream region of the *KLF7* gene transcriptional start site, which contained full-length STAT5 potential binding sites (designated FSPBS), was subcloned into pGL4 luciferase reporter vectors. Additionally, seven truncated mutants (FSPBSΔ1, FSPBSΔ2, FSPBSΔ3, FSPBSΔ1Δ2, FSPBSΔ1Δ3, FSPBSΔ2Δ3, and FSPBSΔ1Δ2Δ3) were subcloned into pGL4 luciferase reporter vectors. All vectors were verified by Sanger sequencing.

Construction of overexpression and knockout HaCaT cell lines

HaCaT cells with *OSMR* or *KLF7* knocked out were generated using the CRISPR/Cas9 system. Briefly, HaCaT cells at 40–50% confluency in 6-well plates were transfected using PEI Transfection Reagent (764604; Sigma, USA) mixed with a plasmid mixture containing 1.5 μg pU6-CAG-Cas9-Puro and 0.5 μg pU6-gRNA-scaffold2.1 (with the DNA oligos encoding the sgRNAs). The transduced cells were then clonally expanded by limiting dilution. Knockout of the target gene products was validated by PCR, fluorescence-activated cell sorting (FACS), or western blotting.

To generate HaCaT cells with p.G513D or p.P694L *OSMR* mutations, recombinant lentiviruses overexpressing *h*OSMR-pG513D or *h*OSMR-pP694L were packaged. A total of 10^5 *OSMR*-knockout HaCaT cells/well were prepared in a 6-well plate. On the following day, the cells in each well were transduced with the packaged recombinant lentiviruses at a multiplicity of infection (MOI) of 100 in DMEM containing 10% FBS with 6 μg/ml Polybrene (TR-1003-GCN; Millipore, Germany). For the controls, lentiviruses overexpressing WT-*OSMR* were used to transduce *OSMR*-knockout HaCaT cells.

To generate the *KLF7*-overexpressing HaCaT cells, *KLF7*-overexpressing recombinant lentiviruses were packaged. A total of 10^5 HaCaT cells/well were prepared in a 6-well plate. On the following day, the cells in each well were transduced with the packaged recombinant lentiviruses at an MOI of 100 in DMEM containing 10% FBS with 6 μg/ml Polybrene.

***In vitro* cytokine stimulation**

Monolayers of HaCaT cells or genetically modified HaCaT cells were washed and maintained 12 h in DMEM with 1% FBS. To measure the phosphorylation of STATs, ERK1/2, or AKT proteins after cytokine stimulation, the cells were treated with OSM (10 ng/ml) or IL-31 (100 ng/ml) for 15 min and then the incubation medium was removed by aspiration. After washing with cold PBS, the cells were lysed for 30 min at 4°C in 200 μl of radioimmunoprecipitation assay (RIPA) lysis buffer (P0013B; Beyotime, China) with a protease inhibitor cocktail (P8340; Sigma, USA). Western blotting was performed to detect the phosphorylation of the proteins.

To detect protein and mRNA expression profiles, HaCaT cells were maintained in DMEM containing 10% FBS, 10 ng/ml OSM, or 100 ng/ml IL-31 for 72 h. 3D skin models were maintained in EpiGrowth medium containing 10% FBS, 10 ng/ml OSM, or 100 ng/ml IL-31 for 72 h. Total mRNA and protein were isolated for quantitative PCR (qPCR), RNA-seq, western blotting, or immunofluorescence analysis.

Small interfering RNA (siRNA)

To downregulate KLF7 expression, 25 pmolof human KLF7 siRNA (GenePharma, China) was used. Two different types of human KLF7 siRNAs were independently assessed for knockdown efficiency (Supplemental Table1). The siRNA was transfected into HaCaT cells using Lipofectamine RNAiMax (13778150; Thermo Fisher, USA) and the transfection medium was replaced after 6 h.

RNA-seq

For the RNA-seq analysis, total RNA samples of HaCaT cells, the epidermis of mice, and patients' tissue were isolated with TRIzol® (15596; Invitrogen, USA). cDNA libraries were prepared using an Illumina/TruSeq Stranded mRNA Library Preparation Kit (RS-122-2102; Illumina, USA). The cDNA was created from 220 pg of input RNA with the SMARTer Ultra Low Input Kit (634848; Clontech; China) and sequenced to a 25-M read depth using Illumina RNA-seq. Reads were aligned to the Ensembl human GRCh38.p13 reference genome or the mouse GRCm38.p6 reference genome with Hisat2 (v2.0.1). SAM files were generated from alignment results using SAM tools. Read counts were obtained with HTSeq (v0.6.1) with the union option. Differential expression was determined using the R/Bioconducter package DESeq2.

Chromatin Immunoprecipitation (ChIP)-qPCR

ChIP assays were performed as previously described (Arcidiacono et al., 2018). Briefly, HaCaT OSM-treated HaCaT or KLF7-overexpressing HaCaT cells were fixed in 1% formaldehyde for 10 min and subsequently quenched with glycine (G8898; Sigma, USA). Genomic DNA was isolated with sodium dodecyl sulfate (SDS) buffer followed by sonication using a Bioruptor (UCD-400; Diagenode, Belgium) to shear the DNA. Immunoprecipitation was performed using antibodies against STAT5 (94205; CST, USA) or IgG (2927; CST, USA). ChIP-grade protein G magnetic beads (9006; CST, USA) were used in the immunoprecipitation and the precipitated DNA fragments were isolated using the ChIP DNA Clean & Concentrator Kit (D5201; Zymo Research, Australia). In the ChIP-qPCR analyses, the values from the immunoprecipitated samples were normalized to that for IgG-immunoprecipitated DNA. The primer sequences are listed in Supplemental Table 1.

EdU incorporation assay

For the *in vivo* proliferation analysis, 5-ethynyl-2'-deoxyuridine (EdU, Sigma, 900584-50MG, USA) was dissolved in sterile PBS to a concentration of 10 mg/ml. A single dose of 50 mg/kg was injected into the mice intraperitoneally 24 h prior to sacrifice.

Histopathology and immunofluorescence

For the immunofluorescence analyses, 3D skins, human skin biopsies or mice skin tissues were submerged in 4% paraformaldehyde for 1 h and then embedded and frozen in optimal cutting temperature (OCT) compound at -80°C. Samples were sectioned at 8-μm thickness, blocked with 2% BSA in PBS/0.3%Triton-X, and labeled with primary antibodies overnight at 4°C. The following day, the unbound primary antibodies were washed off and fluorescence-conjugated secondary antibodies (1:500) were added. Images were acquired on an A1+ confocal microscope (Nikon, USA). For histology, formalin-fixed paraffin-embedded sections of skin were rehydrated in increasingly dilute ethanol concentrations and stained with hematoxylin & eosin or Congo red.

Luciferase reporter assay

Luciferase reporter assays were performed as described previously (Arcidiacono et al., 2018). Briefly, HEK293T cells were pretreated with STAT3, STAT5, ERK1/2, or AKT inhibitors. After 12 h, the cells were stimulated with OSM, transfected with 500 ng of the luciferase reporter plasmid containing full-length or different truncated *KLF7* promoters in 24-well plates. The assays were performed using a Luciferase Assay Kit (E1910; Promega, USA) according to the manufacturer's protocol.

Western blotting

Cells were harvested in RIPA lysis buffer containing a protease inhibitor cocktail. Western blotting was performed as described previously (Liu et al., 2018). The blots were probed with specific primary antibodies against STAT3

(9139; CST, USA), phospho-STAT3 (9145; CST, USA), STAT5 (94205; CST, USA), phospho-STAT5 (9351; CST, USA), AKT (AF6261; Affinity Biosciences, USA), phospho-AKT (AF0016; Affinity Biosciences, USA), ERK1/2 (9102; CST, USA), phospho-ERK1/2 (9106; CST, USA), KRT1 (MA1-35367; Thermo Fisher, USA), KRT10 (NBP2-44707; NovusBiologicals, USA), FLG (NBP1-87528; Novus Biologicals, USA), LOR (ab85679; Abcam, USA), KLF7 (A8879; ABclonal, China), mouse OSMRβ (MAB662; R&D Systems, USA), Myc-Tag (2276; CST, USA), GFP (ab13970; Abcam, USA), and GAPDH (ab181602; Abcam, USA). They were then incubated with horseradish peroxidase-conjugated secondary antibody and the reaction was developed with an enhanced chemiluminescence detection reagent (WBKLS0500; Millipore, USA). The relative band intensity values were measured with ImageJ software. Quantification of protein express levels was calculated as the following formulas: for protein marker, value=[(band intensity value of target protein in the sample)/(band intensity value of GAPDH in the corresponding sample)]/[(band intensity value of target protein in the control sample)/(band intensity value of GAPDH in the control sample)]; for phosphorylated protein, value=[(band intensity value of phosphorylated protein in the sample)/(band intensity value of total protein in the corresponding sample)]/[(band intensity value of phosphorylated protein in the control sample)/(band intensity value of total protein in the control sample)].

Quantitative real-time RT-PCR analysis

Total RNA samples were isolated from cells, 3D skins or mice tissue using TRIzol and then converted into cDNA with a PrimeScript™ RT Reagent Kit (RR047A; TAKARA, China). Quantitative real-time RT-PCR was performed on a LightCycler® 96 Real-Time PCR System (5815916001; Roche, USA) as described previously (Zhang et al., 2018). The primers for the target genes are listed in Supplemental Table 1. The samples were quantified using the comparative $2^{-\triangle\triangle CT}$ method by using human or mouse *GAPDH* as the internal

standard.

Flow cytometry analysis

To identify *OSMR*-knockout HaCaT cell lines, cells were digested and incubated with anti-OSMRβ antibody (AF4389; R&D Systems, USA) for 2 h at room temperature. After washing with cold PBS, the cells were incubated with donkey anti-goat IgG H&L (Alexa Fluor647; ab150131; Abcam, USA).

For the cell cycle analysis, HaCaT cells with or without OSM or IL-31 stimulation were washed with PBS and fixed with 70% ice-cold ethanol at 4°C overnight, followed by treatment with propidium iodide (P4170; Sigma-Aldrich, USA) solution at a final concentration of 50 μg/ml. The DNA content was measured using a flow cytometer (EPICS XL; Beckman Coulter, USA).

Statistical Analysis

The quantitative data were tested for normality using the Shapiro-Wilk test. One-way ANOVA with Bonferroni's multiple comparisons test was used where applicable.

REFERENCES

Arcidiacono, P., Webb, C.M., Brooke, M.A., Zhou, H.Q., Delaney, P.J., Ng, K.E., Blaydon, D.C., Tinker, A., Kelsell, D.P., and Chikh, A. (2018). p63 is a key regulator of iRHOM2 signalling in the keratinocyte stress response. Nature Communications 9.

Huang, H., Zhang, X., Lv, J., Yang, H., Wang, X., Ma, S., Shao, R., Peng, X., Lin, Y., and Rong, Z. (2020). Cell-cell contact-induced gene editing/activation in mammalian cells using a synNotch-CRISPR/Cas9 system. Protein Cell 11, 299-303.

Johansen, C. (2017). Generation and Culturing of Primary Human Keratinocytes from Adult Skin. J Vis Exp.

Liu, J., Li, Q.Q., Li, X.X., Qiu, Z.Z., Li, A., Liang, W.H., Chen, H.Y., Cai, X.S., Chen, X.L., Duan, X.Y., *et al.* (2018). Zika Virus Envelope Protein induces G2/M Cell Cycle Arrest and Apoptosis via an Intrinsic Cell Death Signaling Pathway in Neuroendocrine PC12 Cells. International Journal of Biological Sciences 14, 1099-1108.

Zhang, X., Wang, W., Shan, L., Han, L., Ma, S., Zhang, Y., Hao, B., Lin, Y., and Rong, Z. (2018). Gene activation in human cells using CRISPR/Cpf1-p300 and

CRISPR/Cpf1-SunTag systems. Protein Cell 9, 380-383.

Group	Gender	Age (y)	PLCA type	Sample location
Pat#1	M	32	Macular	Elbow
Pat#2	M	37	Lichen	Calf
Pat#3	F	23	Macular	Calf
Pat#4	F	22	Lichen	Calf

Supplementary Table 1: Demographic and clinical characteristics of the PLCA patients and healthy control involved in this study.

Pat#5	F	21	Macular	Calf
Heal#1	M	21	/	Calf
Heal#2	M	32	/	Calf
Heal#3	F	25	/	Arm
Heal#4	F	30	/	Calf
Heal#5	F	26	/	Calf

Abbreviations: Pat Patient, Heal Healthy control, y years, M Male, F Female.

Supplemental Table 2. Primers, sgRNAs, and siRNAs

Name	Sequence
sgRNA1	TTGTTTAGAATTTTCGTAGT
sgRNA2	GCAGATGTATCCTAATTCCT
sgRNA3	CCAGGTTCTGCATTGGAGCT
sgRNA4	AGGTTGTTTAGACCACCCCA
sgRNA5	CTACAACTTGTCCACGACAC
sgRNA6	CCGTGCATTGAGGAAAGCTT
hKRT1-F	CCGAAGGAGAGTGGACCAAC
hKRT1-R	CTCTGCATTTGTCCGCTTGT
hKRT10-F	ATGTCTGTTCGATACAGCTCAAG
hKRT10-R	CTCCACCAAGGGAGCCTTTG
hFLG-F	TGAAGCCTATGACACCACTGA
hFLG-R	TCCCCTACGCTTTCTTGTCCT
hLOR-F	AGAAGCCATTGAGCTCTCCG
hLOR-R	ACTGGGGTTGGGAGGTAGTT
hGAPDH-F	CACCGTCAAGGCTGAGAACG
hGAPDH-R	GCCCCACTTGATTTTGGAGG
hKLF7-F	TACCATCCCTGGAGGAGACC
hKLF7-R	CCCGAGAGAGCAAGATGTCC
KLF7-ChIP-qPCR-F	TCAGCTCACAGCGCATTTTG
KLF7-ChIP-qPCR-R	GGGGCCTTTACGTGATGAGG
mKrt1-F	GAGCAGATCAAGTCACTCAATGA
mKrt1-R	CCCATTTGGTTTGTAGCACCT
mKrt10-F	GCCTCCTACATGGACAAAGTC
mKrt10-R	GCTTCTCGTACCACTCCTTGA
mFlg-F	ATGTCCGCTCTCCTGGAAAG
mFlg-R	TGGATTCTTCAAGACTGCCTGTA
mLor-F	CTCCTGTGGGTTGTGGAAAGA

mLor-R	TGGAACCACCTCCATAGGAAC
mGapdh-F	AGGTCGGTGTGAACGGATTTG
mGapdh-R	GGGGTCGTTGATGGCAACA
OSMR-E2-F	GATACAGGGAAGGGAGAAG
OSMR-E2-R	CCAAGACTAAGGCAACCAC
OSMR-E3-F	GAGCAAGTCATTTCATTCA
OSMR-E3-R	CTACCAAACCAAAGTTGTC
OSMR-E4-F	CGCTTATTCCACATGAGTC
OSMR-E4-R	TGTTCCCAACCTCTACTCT
OSMR-E5-F	TCAGTGTAACAATAGGAGCAG
OSMR-E5-R	CCCGAAGTAATTCCAGCAT
OSMR-E6-F	AAGGGCAGGAAGGAGAAGG
OSMR-E6-R	AACCCAGATTATCAGACAGGA
OSMR-E7-F	TCCTGTCTGATAATCTGGGTT
OSMR-E7-R	TGAAGTTGGGTAGTAAGCT
OSMR-E8-F	GGCTGTAGTTGGCTCATTC
OSMR-E8-R	ATCTTCAAGGCCCATTTCC
OSMR-E9-F	ACCCTGCTTAATTTTCTAT
OSMR-E9-R	CAACTGTCTGAGGTGGATG
OSMR-E10-F	GTGGACCAGTGGGTAGAAG
OSMR-E10-R	AAAGGAGTGGAGTGGGATT
OSMR-E11-F	ATGCTCTTGAGGCTCTTAT
OSMR-E11-R	GACTGTCCCACTGAAACAC
OSMR-E12-F	TCCTGGTGGTAGTTTGCTG
OSMR-E12-R	ATGCTGGATTTGTCGTTTC
OSMR-E13-F	CCTCTGCCTCTTGGGTTCA
OSMR-E13-R	GATGATTATTCACATAGGGT
OSMR-E14-F	GGGTGTCCATTCTTCATCT
OSMR-E14-R	TTTCCAGTATAAAGCCATG
OSMR-E15-F	TTGTATGCTTTGGCTGGAT
OSMR-E15-R	GGGAGGTGAGAAGGGGAGA
OSMR-E16-F	GGCAGGATAGTATTCTCAC
OSMR-E16-R	TCTTATGGCTAAATGGAGA
OSMR-E17-F	TACTGTATGTTTGGCAATG
OSMR-E17-R	ATTATTAGGTGAGGGTTCT
OSMR-E18-F	AGAGCAGCATCCTGTCATT
OSMR-E18-R	GTGGGAGTCTGTAGAGTGGTT
siCon	UUCUCCGAACGUGUCACGUTT
siKLF7#1	GCUCGGCAGUGGACAUCUUTT
siKLF7#2	GCUCUUCUCUAGACAGCUATT

Figure S1

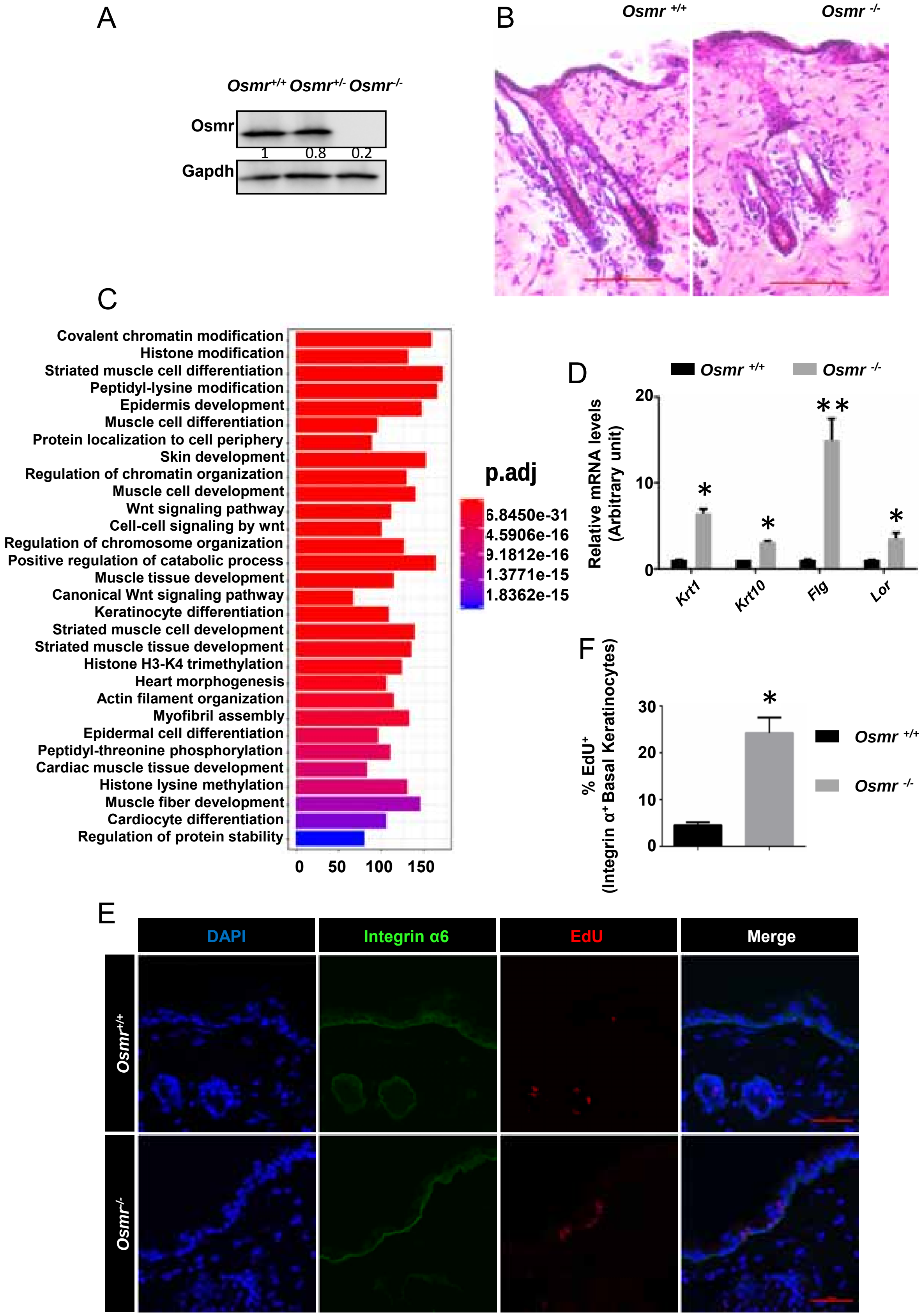

A
Osmr+/+ Osmr+/- Osmr-/-
Osmr
1 0.8 0.2
Gapdh
B
Osmr +/+
Osmr -/-
C
Covalent chromatin modification
Histone modification
Striated muscle cell differentiation
Peptidyl-lysine modification
Epidermis development
Muscle cell differentiation
Protein localization to cell periphery
Skin development
Regulation of chromatin organization
Muscle cell development
Wnt signaling pathway
Cell-cell signaling by wnt
Regulation of chromosome organization
Positive regulation of catabolic process
Muscle tissue development
Canonical Wnt signaling pathway
Keratinocyte differentiation
Striated muscle cell development
Striated muscle tissue development
Histone H3-K4 trimethylation
Heart morphogenesis
Actin filament organization
Myofibril assembly
Epidermal cell differentiation
Peptidyl-threonine phosphorylation
Cardiac muscle tissue development
Histone lysine methylation
Muscle fiber development
Cardiocyte differentiation
Regulation of protein stability
0 50 100 150
p.adj
6.8450e-31
4.5906e-16
9.1812e-16
1.3771e-15
1.8362e-15
D
Osmr +/+
Osmr -/-
Relative mRNA levels (Arbitrary unit)
20
10
0
Krt1
Krt10
Flg
Lor
F
% EdU+ (Integrin α+ Basal Keratinocytes)
30
20
10
0
Osmr +/+
Osmr -/-
E
DAPI
Integrin α6
EdU
Merge
Osmr+/+
Osmr-/-

Supplemental Figure S1, related to Figure 1. (A) Western blot of whole epidermis in *Osmr*$^{-/-}$ mice showing loss of expression of OSMRβ protein. (B) Histological Hematoxylin & Eosin staining of dorsal skin showing differences in hair follicle cycles between WT and *Osmr*$^{-/-}$ mice. Scale bar, 100 μm. (C) Top-ranked enriched Gene Ontology (GO) biological processes in Osmr-knockout mice versus WT mice. (D) qRT-PCR for *Krt1*, *Krt10*, *Flg*, and *Lor* in the epidermis on post-natal day 30 (P30). Data are mean ± SEM, n=3, **p* < 0.05, ***p* < 0.01, one-way analysis of variance (ANOVA). (E) Sections of the dorsal skin from WT or *Osmr*$^{-/-}$ mice after 24 h of EdU incorporation were stained with antibodies against integrin α6 (green) and EdU (red). Scale bar, 50 μm. (F) Quantification of EdU+ basal keratinocytes. Percentages of EdU+ among integrin α6+ keratinocytes. Data are mean ± SD from three independent experiments. **p* < 0.05, one-way analysis of variance (ANOVA).

Figure S2

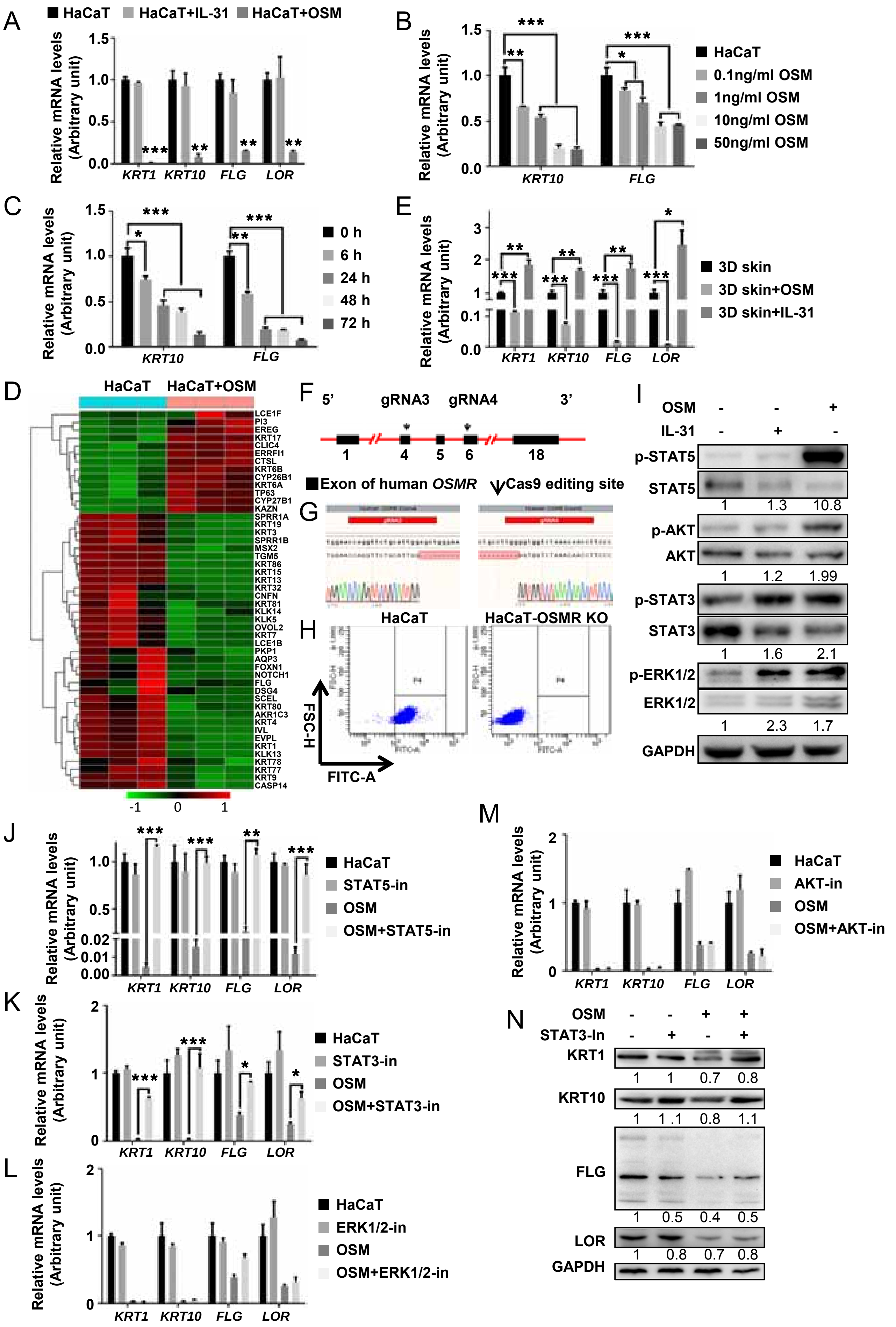

A
HaCaT
HaCaT+IL-31
HaCaT+OSM
Relative mRNA levels (Arbitrary unit)
KRT1 KRT10 FLG LOR
B
HaCaT
0.1ng/ml OSM
1ng/ml OSM
10ng/ml OSM
50ng/ml OSM
KRT10 FLG
C
0 h
6 h
24 h
48 h
72 h
E
3D skin
3D skin+OSM
3D skin+IL-31
D
HaCaT HaCaT+OSM
F
5' gRNA3 gRNA4 3'
Exon of human OSMR
Cas9 editing site
G
H
HaCaT HaCaT-OSMR KO
FSC-H
FITC-A
I
OSM
IL-31
p-STAT5
STAT5
p-AKT
AKT
p-STAT3
STAT3
p-ERK1/2
ERK1/2
GAPDH
J
STAT5-in
OSM+STAT5-in
K
STAT3-in
OSM+STAT3-in
L
ERK1/2-in
OSM+ERK1/2-in
M
AKT-in
OSM+AKT-in
N
STAT3-In
KRT1
KRT10
FLG
LOR
GAPDH

Supplemental Figure S2, related to Figure 2. (A) OSM inhibits *KRT1*, *KRT10*, *FLG*, and *LOR* expression in HaCaT cells. qRT-PCR of HaCaT cells stimulated with 10 ng/ml OSM or 100 ng/ml IL-31 for 72 h. (B) OSM inhibits *KRT10* and *FLG* expression in a dose-dependent manner in HaCaT cells. (C) qRT-PCR analysis *KRT10* and *FLG* expression in different time-points after OSM stimulation. (D) Hierarchical clustering heatmap of differential expression of keratinocyte differentiation-related genes in HaCaT cells with or without OSM stimulation. (E) OSM inhibits *KRT1*, *KRT10*, *FLG*, and *LOR* expression in 3D skins. qRT-PCR of 3D skins stimulated with 10 ng/ml OSM or 100 ng/ml IL-31 for 72 h. (F-H) Construction and identification of *OSMR*-knockout HaCaT cells. Schematic of OSMR-knockout strategy (F), verification of knockout cell clones using Sanger sequencing (G), and flow cytometric analysis of *OSMR*-knockout cells (H). (I) OSM and IL-31 activate different downstream signaling pathways in HaCaT cells. OSM activates the JAK-STAT3/5, AKT, and ERK1/2 pathways, but IL-31 only activates the JAK-STAT3 and ERK1/2 pathways. Western blot of HaCaT cells or HaCaT cells stimulated with 10 ng/ml OSM or 100 ng/ml IL-31 for 15 min. Representative images of three biological replicates. (J-M) qRT-PCR of HaCaT cells stimulated with OSM, with or without pretreatment with inhibitors of STAT5 (J), STAT3 (K), ERK1/2 (L), or AKT (M). (N) Pretreatment with STAT3 inhibitor partially rescues the OSM-induced decreased expression of keratinocyte differentiation-related proteins. Three biological replicates were conducted. For A-C, E and J-M, data are mean $\pm$ SEM, n=3, *$p < 0.05$, **$p < 0.01$, ***$p < 0.001$, one-way analysis of variance (ANOVA).

Figure S3

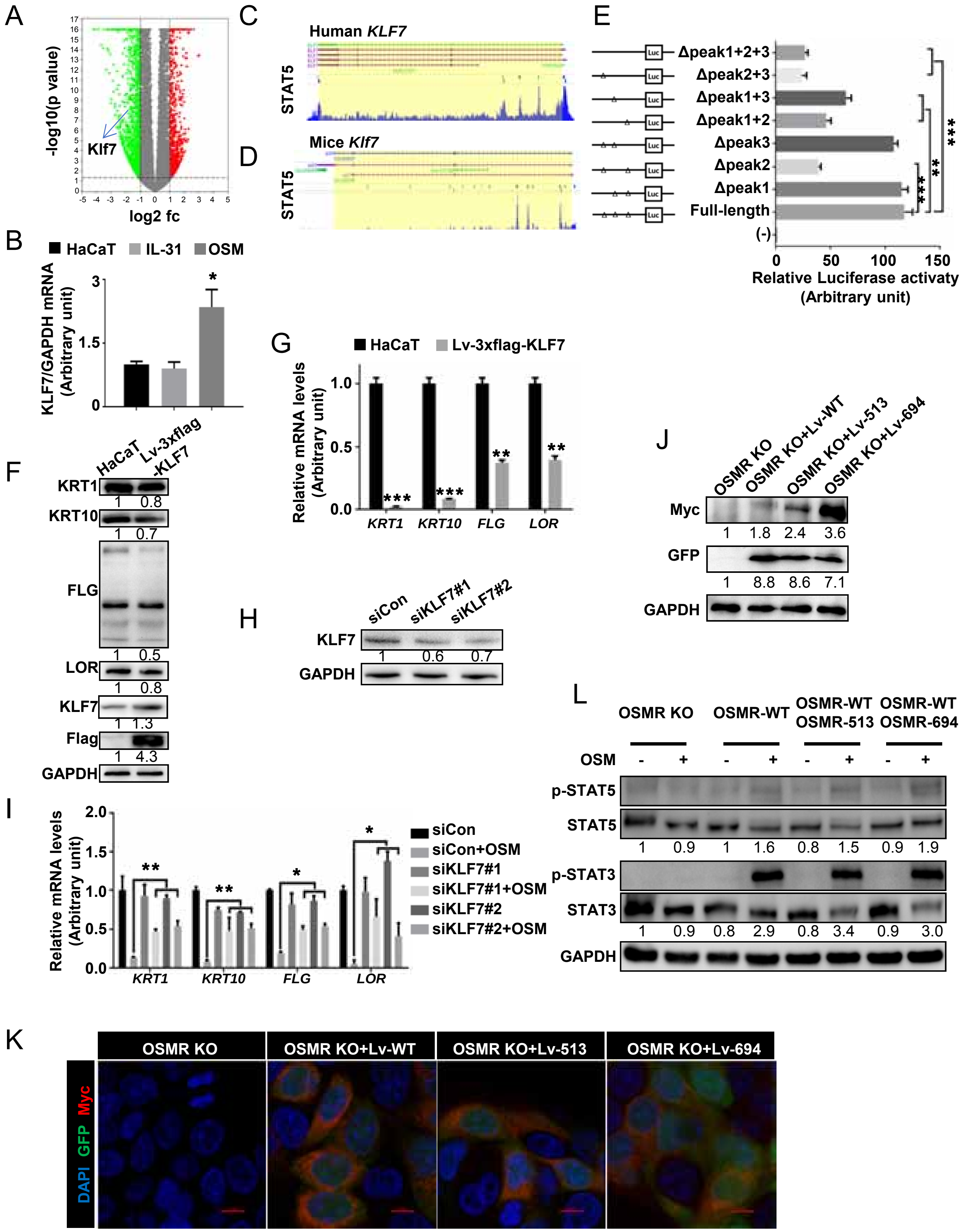

A
-log10(p value)
Klf7
log2 fc
B
HaCaT IL-31 OSM
KLF7/GAPDH mRNA (Arbitrary unit)
C
Human KLF7
STAT5
D
Mice Klf7
STAT5
E
Δpeak1+2+3
Δpeak2+3
Δpeak1+3
Δpeak1+2
Δpeak3
Δpeak2
Δpeak1
Full-length
(-)
Relative Luciferase activaty (Arbitrary unit)
F
HaCaT
Lv-3xflag -KLF7
KRT1
1 0.8
KRT10
1 0.7
FLG
1 0.5
LOR
1 0.8
KLF7
1 1.3
Flag
1 4.3
GAPDH
G
HaCaT Lv-3xflag-KLF7
Relative mRNA levels (Arbitrary unit)
KRT1 KRT10 FLG LOR
H
siCon siKLF7#1 siKLF7#2
KLF7
1 0.6 0.7
GAPDH
I
Relative mRNA levels (Arbitrary unit)
siCon
siCon+OSM
siKLF7#1
siKLF7#1+OSM
siKLF7#2
siKLF7#2+OSM
KRT1 KRT10 FLG LOR
J
OSMR KO
OSMR KO+Lv-WT
OSMR KO+Lv-513
OSMR KO+Lv-694
Myc
1 1.8 2.4 3.6
GFP
1 8.8 8.6 7.1
GAPDH
L
OSMR KO
OSMR-WT
OSMR-WT OSMR-513
OSMR-WT OSMR-694
OSM
p-STAT5
STAT5
1 0.9 1 1.6 0.8 1.5 0.9 1.9
p-STAT3
STAT3
1 0.9 0.8 2.9 0.8 3.4 0.9 3.0
GAPDH
K
OSMR KO
OSMR KO+Lv-WT
OSMR KO+Lv-513
OSMR KO+Lv-694
DAPI GFP Myc

Supplemental Figure S3, related to Figure 2. (A) Volcano plot comparing the p_{adj} value versus fold change for epidermal cells isolated from WT and *Osmr*$^{-/-}$ mice. Green and red dots represent genes with a p_{adj}<0.05 and >2-fold. (B) qRT-PCR of HaCaT cells stimulated with OSM or IL-31. (C and D) Screenshot of the WashU Epigenome Browser from chromatin immunoprecipitation (ChIP)-sequencing analysis of human B lymphocytes (C) and mice natural killer cells (D) with an anti-STAT5 antibody. (E) Luciferase reporter assay of the *KLF7* promoter with mutated STAT5 binding sites. The luciferase reporters were transiently transfected into HEK293T cells and treated with OSM. (F) Lentivirus-induced KLF7 overexpression in HaCaT cells decreases the expression of keratinocyte differentiation-related proteins. (G) qRT-PCR of indicated genes in HaCaT cells and KLF7-overexpressing HaCaT cells. (H) Western blot showing KLF7 expression in HaCaT cells treated with the control siRNA (non-targeted protein, NTP) or KLF7 siRNAs. (I) Knockdown of *KLF7* in HaCaT cells partially rescues the OSM-induced decreased expression of keratinocyte differentiation-related genes. (J) Western blot (anti-Myc) showing expression of recombinant WT and mutated *OSMRs* in HaCaT cells. (K) Immunofluorescence analysis showing the cytoplasm and cell membrane localization of the recombinant proteins (Myc). Scale bar, 10 μm. (L) No dominant negative effect was observed in *OSMR* p.G513D or p.P694L mutant HaCaT cells. Western blot of OSMR-knockout HaCaT cells transduced with indicated OSMR mutants. For B, E, G, and I, data are mean ± SEM, n=3, *p < 0.05, **p < 0.01, ***p < 0.001, one-way analysis of variance (ANOVA).

Figure S4

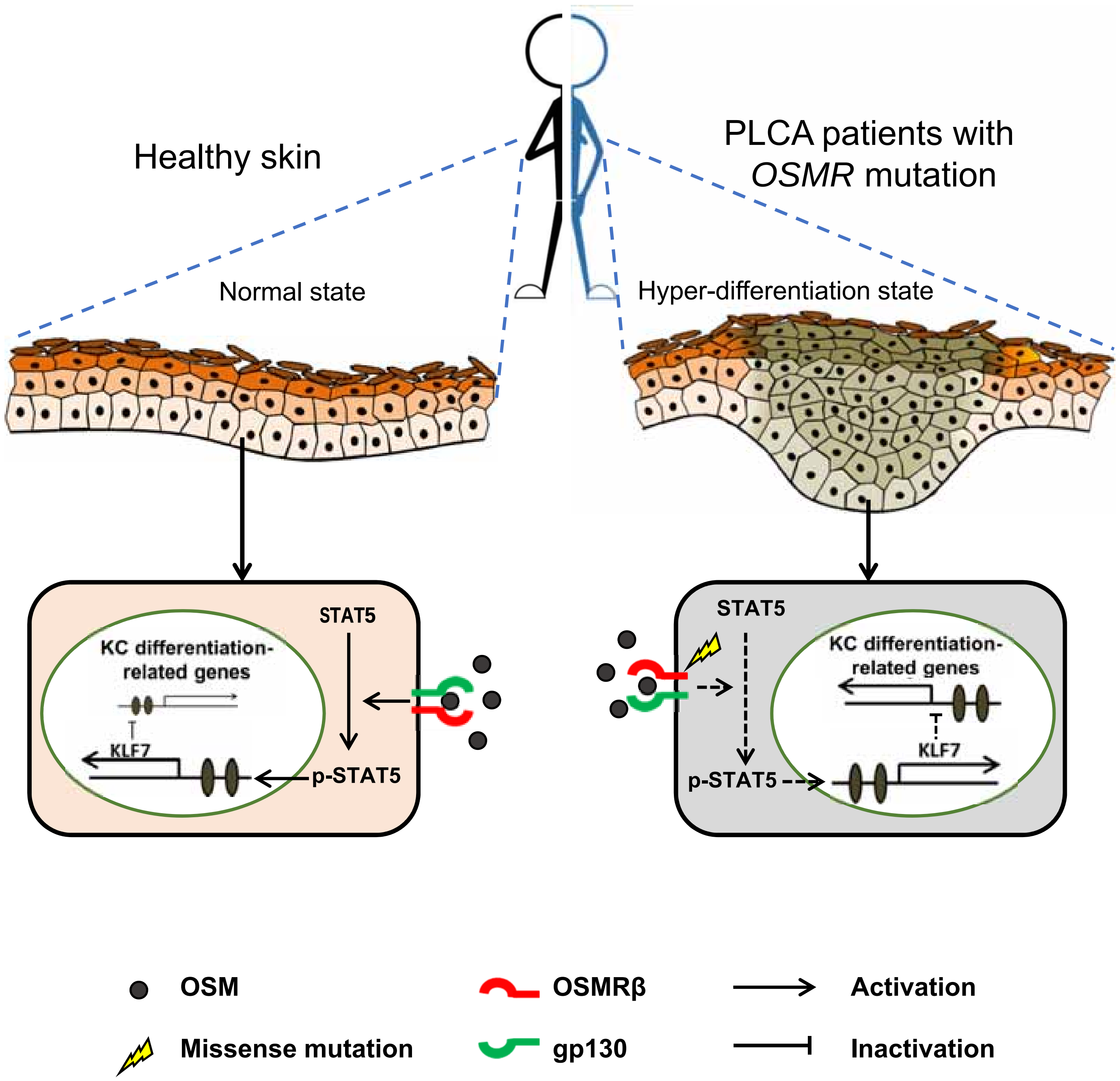

Healthy skin
PLCA patients with
OSMR mutation
Normal state
Hyper-differentiation state
STAT5
KC differentiation-related genes
KLF7
p-STAT5
STAT5
KC differentiation-related genes
KLF7
p-STAT5
OSM
OSMRβ
Activation
Missense mutation
gp130
Inactivation

Figure S4. Schematic model of *OSMR* mutation-induced basal keratinocyte differentiation. In physiological condition, OSM binds to OSMR on the keratinocyte cell membrane and activates STAT5 in the cytosol, and STAT5 translocates to nucleus and activates KLF7, which inhibits the expression of key genes of keratinocyte differentiation. In pathological condition of PLCA patients with loss-of-function *OSMR* mutants, OSM is unable to phosphorylate STAT5 and activate KLF7, and fails to maintain a low expression level of key genes of keratinocyte differentiation.
